# Understanding paramedic work in general practice in the UK: a rapid realist synthesis

**DOI:** 10.1186/s12875-024-02271-1

**Published:** 2024-01-23

**Authors:** Hannah Stott, Trudy Goodenough, Justin Jagosh, Andy Gibson, Nicky Harris, Cathy Liddiard, Alyesha Proctor, Behnaz Schofield, Nicola Walsh, Matthew Booker, Sarah Voss

**Affiliations:** 1 University of the West of England, Centre for Health and Clinical Research, Glenside Campus, Blackberry Hill, Bristol, BS16 1DD UK; 2 Centre for Advancement in Realist Evaluation and Synthesis (CARES), Vancouver, Canada; 3 Bristol Medical School, University of Bristol, Centre for Academic Primary Care, Bristol, BS8 2PS UK

**Keywords:** Paramedics, General practice, Primary health care, Health workforce, Realist synthesis

## Abstract

**Background:**

General practice in the UK is under substantial pressure and practices are increasingly including paramedics as part of their workforce. Little is known about how different models of paramedic working may affect successful implementation of the role, as viewed from patient, clinician and system perspectives. This realist synthesis developed theories about ‘models of paramedic working in general practice’ in different UK contexts to understand their impact.

**Methods:**

The rapid realist synthesis comprised data from: (1) empirical and grey literature searches; (2) semi-structured realist interviews with system leaders involved with the implementation of the role; and (3) a stakeholder event with healthcare professionals and the public, to develop initial programme theories that can be tested in future work. Sources were analysed using a realist approach that explored the data for novel or causal insights to generate initial programme theories.

**Results:**

Empirical sources (*n* = 32), grey sources (*n* = 95), transcripts from system leader interviews (*n* = 7) and audio summaries from the stakeholder event (*n* = 22 participants) were synthesised into a single narrative document. The findings confirmed the presence of a wide variety of models of paramedic working in UK general practice. The perceived success of models was influenced by the extent to which the paramedic service was mature and embedded in practice, and according to four theory areas: (1) Primary care staff understanding and acceptance of the paramedic role; (2) Paramedic induction process, including access to training, supervision and development opportunities; (3) Patient understanding and acceptance of the role; (4) Variations in paramedic employment models.

**Conclusions:**

Variability in how the paramedic role is operating and embedding into general practice across the UK affects the success of the role. These findings provide a theoretical foundation for future research to investigate various ‘models of paramedic working’ in different contexts.

**Supplementary Information:**

The online version contains supplementary material available at 10.1186/s12875-024-02271-1.

## Background

General Practice (GP) services are under increasing pressure due to a growing and ageing population and increasing healthcare demand [1–3]. Many GP services are also providing alternative access to urgent care to alleviate demand on Emergency Departments and move care closer to home [4]. However, such services are struggling to meet these demands due to a shortage of GPs and a lack of funding [5, 6]. As a result, GP services are evolving to increase the non-medical workforce supporting front-line service delivery [5], and additional funding is being allocated for these roles [7].

Paramedics are one group of allied health professionals working in general practice in increasing numbers [8], whose generalist skillset may be well-suited to this setting. Previous research indicates that paramedics carry out a range of tasks in general practice including home visits, routine appointments, same-day clinics and telephone triage. There is significant variation in the types of condition and patient groups that paramedics are employed to manage [9] and in the model of contractual engagement, including rotational schemes where some clinical time in the ambulance service is retained [10].

Practice service requirements and patient populations create further variation in how the paramedic role is utilised, as will the qualification and experience of the paramedic entering the role. For example, in 2018 legislation to permit paramedic prescribing was enacted, enabling them to potentially manage more patients autonomously.

Paramedics have been working in general practice since at least 2002 [11], but there are few studies which describe how the role operates and in what ways it contributes clinically (and otherwise) in this setting. Whilst patient acceptance of the role has been explored [12], much of the remaining literature focuses on which extended skills are needed by paramedics in order to work autonomously or safely in primary care [13–16]. This research is largely descriptive and includes many assumptions, such as paramedics reducing GP workload and costs, which have not been tested empirically. A further assumption is that it is cost effective to employ paramedics; however, they may need substantially more time than GPs to assess and treat patients, require considerable support in terms of induction and supervision and may make different clinical decisions which may affect subsequent health resource use.

A recent realist review [17] to understand the role of paramedics in primary care focusses on the perspective of the paramedic, discussing role boundaries and professional identity. This review concludes that formal education and clinical supervision are key to the successful transition of paramedics into general practice. However, to date, no literature review examines the variation in paramedic models of working in general practice and the associated impacts on patient safety, clinical or cost effectiveness. Implementation guidance struggles to reflect this variation, making it difficult for practices to make informed decisions around how to successfully implement the paramedic role in their specific context. The aim of this rapid realist synthesis is to describe the different ways paramedics are working in general practice in the UK and explain how variation may impact on clinical and cost effectiveness of the role.

## Methods

Realist methodology provided a suitable approach for understanding the complexity of the paramedic role in general practice, and its associated outcomes. Due to the variation in working arrangements for paramedics in general practice, it was not appropriate to conduct a traditional systematic review to determine if their involvement was effective or not. Instead, the realist approach was used to ask: “What it is about models of paramedic working in general practice that works, for whom, in what circumstances and how?” [18]. Realist methodology answers these questions by developing theories to illustrate how an intervention can lead to a variety of intended and unintended outcomes. These theories clarify how active mechanisms are affected by the context in which they are introduced, and these relationships provide causal explanations for observed outcomes, illustrated as context-mechanism-outcome configurations [19]. Mechanisms can be separated into resources (provided by the intervention) and reasoning (the ways in which this changes the response of stakeholders) [20].

In order to produce findings that are responsive to a rapidly changing NHS workforce, we opted to conduct a rapid realist review and supplement data from the literature with interviews conducted with key stakeholders. Rapid realist reviews are designed so that researchers can engage with key stakeholders and knowledge experts on the research questions; this enables the review process to be streamlined. [21]. This review was written to RAMESES publication standards [22].

The synthesis was designed and conducted in collaboration with a realist methods expert (JJ), a large interdisciplinary team (*n* = 19) and a group of public contributors (*n* = 7) who were trained in realist methodology.

The realist approach permitted the inclusion of empirical and non-empirical literature (see Table 1 for data sources), reflecting the most up-to-date information describing different models of paramedics’ work. Data collection and analysis contributed insights into how and why these models achieve different outcomes in different settings, as listed below.

**Table 1:** Data sources for rapid realist review

Data source	Details
Empirical literature	See additional file 1 for details of literature search
	See additional file 4 for empirical literature included in review
Grey literature	See additional file 2 for search terms
System leader interviews	Interviews with leaders involved with the implementation of paramedics in general practice
	See additional file 3 for interview schedule
Stakeholder event	Involving professional and public stakeholders, to clarify areas of priority and identify any gaps in theory development

### Empirical literature

An empirical literature search was completed in July 2021 in 8 databases: Medline, AMED, CINAHL Plus, PsychINFO, British Nursing Database, Embase, Cochrane Library (CCRCT & CDSR), and Scopus. These databases were selected in consultation with an information specialist in health research and agreed by the study team to be a sufficient number of databases to minimise selection bias. To ensure a broad and comprehensive search, the search terms linked synonyms of the term ‘general practice’ and ‘paramedic’ (Additional File 1). Where possible the date limiter ‘after 2002’ was utilised as this is when the paramedic role in general practice was first established in the UK [23]. Papers were included if they mentioned in the title or abstract synonyms of ‘paramedic’ and ‘general practice’.

Records were initially screened on title and abstract by one of three authors (HS, JJ, SV). A second author (NH) dual screened 55% of these papers. Exclusion criteria were iteratively refined as more was understood about the literature, which led to a two-phase screening process.

In phase one, papers were excluded if they were published before 2002 and if they focused on a non-UK setting as we wanted to develop theory relevant to the unique NHS context. In phase two, papers were excluded if they focused on paramedic roles outside of general practice (e.g. in community, out of hours and emergency settings), or if they primarily focused on other allied health professions. Initial included papers were re-examined in the second phase of screening on title, abstract and, where necessary, full text.

Records identified in the searches were exported into Covidence software for screening and duplicates were removed automatically. A subgroup of excluded papers, termed ‘transferable findings’, were earmarked for later consideration because they described other roles in general practice which may provide relevant insights to the paramedic role in a UK general practice setting.

### Grey literature

Grey literature searches were completed between Dec 2021 and Jan 2022 in: two social media platforms (Twitter and Facebook); one video-sharing website (YouTube); one search engine (Google); and seven health professional websites using search parameters appropriate to the data type (Additional file 2). Searches utilised the terms ‘paramedic’ AND ‘primary care’ OR ‘general practice’. The same exclusion criteria were used as in the empirical search. Two researchers (HS, TG) screened titles and summaries, and identified eligible sources.

### System leader interviews

Semi-structured interviews were conducted with purposively sampled participants with a leadership role on the implementation of the role of paramedics in general practice between Sept and Nov 2021. The target sample size was 8–10 participants in line with the concept of information power for a study with expert participants investigating a narrow focus (ref) [24]. Participants were employees of four national organisations: The College of Paramedics (CoP); The Department of Health and Social Care (DHSC)​; Health Education England (HEE)​, and NHS England​ (NHSE). Participant perspectives represented their personal viewpoints, not those of their affiliated organisation. Potential participants were identified via consultation between the study team and Steering Committee who identified known contacts or networks to approach. Once participants assented to contact, study information was shared with them directly and they provided written informed consent prior to interview. Participant information sheets explained that all data would be stored and processed in in accordance with General Data Protection Regulation (GDPR) along with the Data Protection Act 2018 (DPA). All data would be anonymised and no participant would be personally identifiable from any reports or outputs from the research. Participants were informed of their right to withdraw from the study up until the point that their data had been anonymised and entered into the analysis. Interviews were conducted using an online video platform by two interviewers (HS and TG). Audio was recorded and transcribed by an external provider. The interview schedule is shown in Additional file 3.

### Stakeholder event

A stakeholder event was held with a group of professional and public stakeholders with interest or experience of the role of paramedics in general practice. The purpose of the event was to present and discuss the findings from the literature review and interviews and generate early consensus on the relevance of the emerging insights. Participants were recruited by promoting details of the event on social media, and through directly contacting stakeholders via email who previously expressed an interest in taking part in the event and had assented to further contact. Participants who registered to take part were emailed a participant information sheet and consent form. The information sheet explained confidentiality, anonymity and participants’ right to withdraw from the research. All participants provided written informed consent.

The event was hosted using an online video platform. Study information and early findings from the review were presented in stages and participants had opportunities to discuss each topic using verbal and instant message chat. Participants were also provided with a list of statements which focused on the variation between different service models and how this variation may impact outcomes, which they discussed and then indicated areas they perceived to be important. A public engagement facilitator (AG) facilitated balanced discussion between professional and public stakeholders. The discussions were noted throughout the meeting and presented back to stakeholders on slides at the end of the session, to ensure agreement of the points discussed.

### Analysis and synthesis

All data were analysed using a realist technique called ‘appraisal journaling’. This technique appraised evidence by highlighting and then expanding on highly relevant passages that provided key causal insight into contexts or mechanisms operating in the programme of interest [18]. The researchers familiarised themselves with the data and then captured insights by providing a brief summary, followed by journaling their evidence-inspired reflections on the concepts of interest and realist context-mechanism-outcome configurations. This process provided space to record well-evidenced theory alongside novel concepts, consider the plausibility of these new ideas, and identify gaps in theory [25].

The full text of the empirical papers, abstracts and articles were analysed in reverse chronological order, starting with the most recently published papers. Five members of the research team (JJ, HS, TG, SV, MB) engaged in the ‘appraisal journaling’ process. Reflections on each paper were captured in order, using one Word document to capture and share ideas. Five of these papers were also analysed by the team of public contributors to provide a public perspective on the role [Additional File 4: Papers 2, 3, 11, 22, 27]. The excluded papers with transferable findings were reviewed by one team member (JJ) and relevant insights were noted.

Following this, the remaining data sources were explored. Each data type was analysed individually, and as the data was extracted into each Word document, it was filed or grouped inductively into concepts of interest or themes, using headings in Word. The data under each of these topic themes across the three remaining data sources were then explored using the ‘appraisal journaling’ process. The grey literature was examined by two authors (HS, TG), and relevant data extracted into a separate Word document. Then, the system leader interview transcripts were read, analysed thematically and initial themes were discussed as a team (JJ, HS, TG, SV, MB, BS). Relevant data were directly extracted into a further Word document by one author (HS), in the order in which the interviews were conducted (CoP, DHSC, HEE, NHSE). Finally, the audio recording from the stakeholder event appraised by two authors (HS, TG) and relevant insights were transcribed and extracted into a final Word document.

To synthesise the findings, one author with extensive experience in realist methodology (JJ) reviewed the analysis documents and drew together the key insights into a single narrative document. Analysis of the empirical literature provided a framework of ideas around which to build the synthesis, and reflections on the grey literature, system leader interviews and stakeholder event were incorporated into this framework. The process was conducted iteratively, in that drafts of the synthesis were shared with the study team and Patient and Public Involvement (PPI) contributors (who were not research participants or respondents) during development to allow feedback and discussion among the team about the concepts being highlighted and to ensure the synthesis accurately reflected the data.

Realist methods encompass subjective reasoning to draw causal links between claims, which allows researchers to theorise more deeply as to how mechanisms of paramedics working in general practice are influenced by different contexts to create varied outcomes. In this review any subjective reasoning was discussed within the research team to understand the issues from a variety of perspectives and to ensure the most plausible theory was selected. It should be noted that whilst these theoretical claims are derived from data, they were not lifted verbatim from single sources, but rather synthesised from a variety of sources to develop theories.

## Results

### Empirical and grey literature

**Fig. 1:**
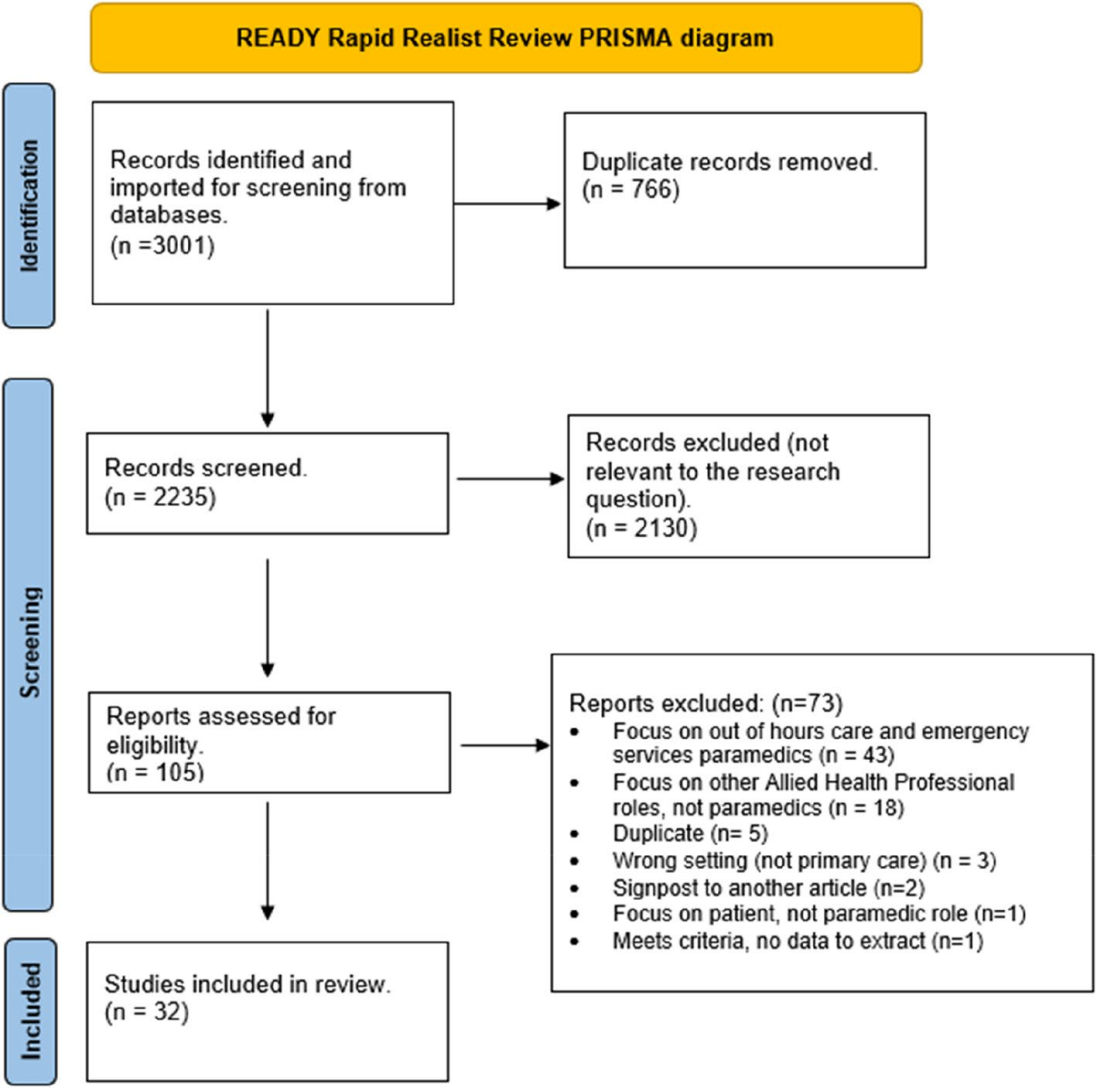
PRISMA Empirical document selection and appraisal flow diagram

The empirical literature search returned *n* = 3001 papers. Duplicates (*n* = 766) were removed. Records for *n* = 2235 papers were screened on title and abstract and *n* = 105 papers were included after initial screening. Consensus on exclusion for 55% of the records that were dual screened was 100%. During the second phase of screening *n* = 73 were excluded, leaving *n* = 32 papers for analysis (Fig. 1. PRISMA; Additional File 4). There were *n* = 11 papers in the subgroup of excluded papers termed ‘transferable findings’. One particularly insightful article [17] which reviewed 205 papers on the role of paramedics in general practice was searched to identify any additional sources. One additional empirical source was identified [26] and included within this subgroup of transferable findings (*n* = 12; Additional file 4). The grey literature search identified *n* = 431 eligible sources and after screening, *n* = 95 were included in the analysis. Papers were included based on richness and relevance of the data to the research questions. [22]

### System leader interviews

Seven semi-structured interviews were conducted with 8 participants (one interview included two participants). Interviews lasted between 32 and 59 min and the mean interview length was 39 min.

#### Stakeholder event

The stakeholder event was two hours long and was attended by 22 participants, made up of 14 professionals (including paramedics in general practice, GPs, and professionals with leadership roles in the delivery of urgent and primary care) and 8 public contributors.

### Synthesis

The synthesis encompassed four theory areas, as illustrated in Table 2. The data sources which contributed to the development of these areas are shown in Additional file 5. Findings indicated there was considerable variation amongst the models of implementation of the paramedic role: paramedics were working under different employment models; in new or more mature and embedded roles; and there was wide variation in qualifications and types of patients and conditions seen. These concepts were evident across the four theory areas. Each theory area is discussed below with key literature references or stakeholder quotes, followed by provisional CMO configurations.

**Table 2:** Summary of provisional theory areas

Heading	Provisional Theory Area
**Primary Care Staff Understanding of the role of the paramedic in general practice**	1. Understanding “Advanced Paramedic Practice”
	2. Ensuring the “right fit” for home visits
**Paramedic Induction Process, and access to training and development opportunities**	3. Education and Training at Induction
	4. Ongoing supervision and training of paramedics in general practice
**Patient Understanding and Acceptance of the paramedic role in general practice**	5. Patient Perceptions of paramedic role
**Variation in Paramedic Employment Models**	6. Benefits of Rotational Models for paramedics working in primary care

- [1] Primary care staff understanding of the paramedic role in general practice

Paramedics are attractive to general practice because of their professional culture of innovating and problem solving and there is an expectation that they can provide autonomous generalist care [27]. There was a lack of clarity amongst both paramedic staff and general practice about the meaning of the term ‘advanced practice’ (level 7) in general practice [28], *“which generates huge amounts of challenges because people say ‘I'm an advanced paramedic’ and they’ve got no level seven study at all.” (System leader interview 3).* However, paramedic skillsets were developed to meet the requirements of urgent and emergency contexts; the misperception of the role by staff in general practice led to difficulties matching paramedic skillsets to the patient population.

*“Primary Care don’t get it. They think that paramedics can work in the same way that nurses can, [but] they’re not trained to do minor illnesses, to manage frailty… there’s litigation cases, across the country, for unsafe practice… they [GPs] are not understanding what a paramedic can bring, I think that maybe paramedics are being put into the wrong space sometimes” [System leader interview, No.4]*

CMO 1 Understanding advanced paramedic practice and ensuring ‘right fit’.

**Table:** 

Context: Lack of consistency in the understanding of the term “Advanced Practitioner” by paramedics and general practice staff
Mechanism: Paramedics are asked to see patients (resource) whose problems may be outside their skillset, and they are unprepared to deal with the clinical challenges they see (response)
Outcome: for the paramedic: uncertainty about role, remit and capabilities for the patient: suboptimal care for the practice: risk of unsafe practice, medical error, and litigation

Paramedics required support when transitioning from urgent to primary care as they developed more complex and autonomous clinical reasoning skills, in contrast to a reliance on protocol-driven decision-making in the ambulance service [29].

“*When employing junior paramedics in a primary care setting it is important for both parties to understand and appreciate that exposure to the complexity and intensity of primary care should be undertaken gradually and at a rate which is comfortable for both parties as well as safe for patients”* [30], p24].

There was consensus that when entering general practice, paramedics needed to clarify the scope of their role, in terms of the types of patients and conditions they are able to manage.

*“there’s probably a bell curve distribution of practice for paramedics… Some are providing excellent care that is in line with advanced level clinicians within primary care, most are providing safe care with support and supervision from GPs, but I imagine there will be a section of the paramedics out there that are providing care that is probably unknowingly out of their scope or might not be safe, or as safe as it could be if they had more education and qualifications” (Paramedic, Stakeholder event).*

Paramedics’ capacity to conduct home visits provided a beneficial extension of general practice services to patients’ homes [17]. This model suited GPs and paramedics, because home visits were considered time consuming, and paramedics were considered experts in community health care [31]. Reducing the GP role in home visits was thought to free up time and increase GP appointment capacity in practice [32], although supervising home visits for less experienced paramedics to ensure safe provision of care by staff unfamiliar with the management of frailty or complex co-morbidity did generate a workload burden for GPs (GP, Stakeholder event; [9]). Concern was also expressed that altering role boundaries may lead to GPs losing home visiting skills (GP, Stakeholder event), or compromising continuity of care [33].

CMO 2 Ensuring paramedic ‘right fit’ for home visits.

**Table:** 

Context: Home visits are time consuming for GPs, but inexperienced paramedics or those with fewer post-registration qualifications may be unable to manage some home visits autonomously
Mechanism: Provision of remote support and supervision from the GP (resource) supports safe practice and provides the paramedic, GP and patient with reassurance (response) about standards of care provided
Outcome: For patient: Timely home visit For paramedic: Supervision whilst gaining experience and developing skills For GP: Saving time. Initially any time saved by not doing visits may be consumed by supervision whilst a paramedic home visit service is embedding, but supervision requirements should reduce over time

Paramedic home visiting improved timely access to care for patients by increasing the capacity for morning appointments (NHS England, Beacon Medical case study [34]) creating potential benefits for patient outcomes, patient satisfaction and reduced conveyance to hospital.

*“Utilising specialist paramedics to undertake home visits earlier in the day will smooth the flow of primary care home visiting activity which typically occurs around lunchtime when GPs finish morning surgery”* [35].

- [2] Paramedic Induction Process, including access to training, supervision and development opportunities

The transition from working in ambulance roles to working safely and autonomously in general practice was more successful when paramedics had access to training and development opportunities from the outset. This was because specific skills required for general practice roles were beyond the scope of ambulance paramedics’ core capabilities, such as *“the routine management of multimorbidity and chronic disease, a shift towards preventative care, and a mastery of more nebulous concepts as ‘continuity’ and ‘the therapeutic consultation.*’” [28].

Access to training was also a key driver in attracting paramedics to the role, and in retaining them by ensuring their role was challenging and varied.

*“More training, prescribing for example, because they are also given protected time to do that learning… what we have seen is Paramedics starting to go back to Ambulance Trusts because after a while, if the practice don’t utilise them to their maximum potential, they get bored” [System leader interview 5].*

Requirements for paramedics in general practice to attain ‘Advanced practice’ qualifications via Health Education England accreditation were becoming accepted as a standard, though academically demanding, part of paramedic development in general practice [Social media, 2021].

General practice highlighted the need for development of specific paramedic skills, such as interpretation of blood tests which would support paramedic knowledge around prescribing [30, 36]. Yet it was also acknowledged that paramedics brought new skills to general practice for example in triage, minor injury treatment, catheter management and emergency care which reduced pressure on duty doctors and other primary care staff [37, 38].

CMO 3 Education and training mechanisms at paramedic induction into general practice.

**Table:** 

Context: Paramedic formal training typically does not include routine management of many medical conditions, or managing multimorbidity or chronic illness, but paramedics in general practice may need to diagnose and treat patients experiencing these conditions
Mechanism: Providing the time, resources and support for paramedics to undertake training allows paramedics to gain critical pathophysiological knowledge to treat patients in general practice (resource) which develops paramedic clinical skills and confidence to manage these patients autonomously (response)
Outcome: For patient: improved safety and standards of care For paramedic: Improved clinical decision-making; reduced need for intensive, time-consuming supervision For practice: Improved retention of paramedics in primary care

There was wide variation in the degree to which paramedics could practice autonomously and confidently (e.g. conducting advanced clinical decision making and using skills such as prescribing) within a general practice patient population. This affected the scope of paramedic workload and the workload of other general practice staff. For example, some paramedics were *“consulting with GPs on almost a case-by-case daily basis, to use them as consultants and prescribers”* (Paramedic, stakeholder event), whereas other paramedics were *“leading on frailty… will help run the emergency clinic, they’ll have their own consulting room and actually go through the patients on the emergency list in the morning alongside the GP”.* (Education provider, stakeholder event).

Quality supervision was considered key to successful and safe implementation of the role [36], however the addition of supervisory tasks added to the workload of GPs and other staff responsible for this role and matching the skillsets of supervisors to paramedics was a challenge.

*“for a lot of the PCNs (Primary Care Networks), (supervision) is also an issue, because they don’t know how best to support the roles. You can’t have this brand new, huge new workforce, and expect the GPs to do all the supervision because that just adds to their workload and they’re not necessarily the right people to be doing it either…”. (System leader interview 6)*

CMO 4 Routine supervision of the paramedic role.

**Table:** 

Context: Paramedics entering general practice roles have a variety of skillsets and experience which affect their ability to work autonomously
Mechanism: Routine supervision of paramedics by appropriate practice staff, especially at the outset of the role, offers the opportunity to have enhanced discussions about patient care (resource), which will help to inform practice staff about paramedic scope, and will clarify appropriate ways for the paramedic to manage patient care (response)
Outcome: For patients: Improved patient outcomes and safety For paramedics: Improved staff satisfaction For practice: Increased GP supervision workload whilst embedding the role Over time, the paramedics general practice skillset and ability to work autonomously should develop, and the supervisory burden may reduce

- [3] Patient acceptance and understanding of the role

Patients generally appeared to be satisfied with the paramedic role in primary care. A small-scale survey of *n* = 80 patients who were treated by a general practice paramedic reported being happy (73%) or very happy (18%) with their experience [39]. Whilst GP home visits typically occurred after morning surgery, introducing paramedics enabled patients to receive home visits earlier in the day [40].

However, a qualitative study with six patients reported a lack of patient clarity about who was conducting the home visit: *“At all times, the participants were expecting a GP. Despite being told that they were seeing a PP [paramedic practitioner], participants repeatedly said ‘thank you doctor’ at the end of the consultation.”* [12]. This confusion about the role may have implications for patients who primarily associate paramedics with urgent responses to serious conditions.

*“Patients held preconceived ideas about the role of ambulance service staff, and that the arrival of an ECP [emergency care practitioner] meant they were sufficiently unwell to require hospitalisation. Reception staff aimed to explain to patients that the home visit may be performed by an ECP. However, many patients reported being unaware that an ECP would arrive.”* [41], p71.]

CMO 5 Patient perceptions of the general practice paramedic role.

**Table:** 

Context: Patients and the public have a traditional view of the paramedic being solely involved in emergency care
Mechanism: Patients have opportunities to see paramedics in non-emergency roles during home visits and booked appointments (resource), and opportunities to discuss questions about the nature of this role with reception staff (resource), leading to a revised view of the role of paramedics (response) and increased clarity and confidence about their role in general practice
Outcome: For patients: Timely, effective clinical care; increasing exposure to paramedic-led care normalises the role for patients For paramedics: Increasing levels of patient acceptance For practice: GPs have more time to attend to more complex patients, and the practice deals with fewer patient concerns about “not seeing a GP”

Patients showed some concern that paramedics’ skills were not equivalent to a GP’s skill, but this was less of a problem if they felt their symptoms fitted within their perception of the paramedic scope or if it was not an ongoing condition. *“I would prefer to be seen by a GP obviously, but it depends on the reason, if I had anything that a paramedic could deal with, then that would be absolutely fine.’* [12, p 119]*.* However, patients had limited understanding of what the paramedic skillset and scope involved [12]. Two participants in the stakeholder event stated that they would prefer to see a paramedic for certain conditions because *“they have more experience in crisis management… they are less judgmental.” [Public contributor 1].*

Many patients were supportive of the need to lessen the load on GP staff by utilising the paramedic workforce: *‘I would be quite happy to see the PP [paramedic practitioner] than waiting longer to see the GP, as I see it, it’s obviously a way of reducing the pressure on the surgeries which I can understand.’* [12, p 118].

- [4] Variations in paramedic employment models in general practice

Paramedics were employed in general practice under a variety of employment models depending on location, cost or general practice requirements, which had implications for how the role operated in different settings.

*“across the country we have rotational models and we have substantively employed models…Primary care quite like that [substantive] model because… they own that person and they're part of that family and they can help and support and develop them.” [System leader, interview 3].*

Rotational models involved paramedics working in primary care at regular intervals while retaining their role in emergency services. Paramedics in these roles were likely employed by ambulance trusts or Primary Care Networks.

*“Paramedics on a rotational model worked really well in some parts of the country…they are looking at it as a sustainable business model, whereas in other parts they’ve looked at it purely as a staff retention model. So, they haven't made money out of it but they’ve retained staff.” [System leader, interview 3]*

The variety of tasks associated with a rotational employment model (managing the diverse needs of both patients who require Emergency Medical Services and those who contact general practice) were thought to be beneficial for the development of clinical skills and autonomy, and paramedic staff retention [42]. This is seen as advantageous to both general practice and ambulance services [43].

CMO 6 Benefits of Rotational Models of paramedic working.

**Table:** 

Context: Paramedics working in traditional ambulance roles defer to guidelines to determine whether a patient should be admitted to hospital or not, and transfer care to other clinicians to make decisions around ongoing management. Decision-making and risk management is a more binary and immediate process, unlike longer-term management options in primary care
Mechanism: Rotational employment which includes work in the home visit setting provides exposure to a wider array of presentations in the primary care patient population compared to attendance for emergencies (resource). Supervision by GPs (resource) allows paramedics to develop clinical autonomy and an awareness of longer-term management options that are alternatives to hospital admission (response)
Outcome: For patient: Care can be personalised rather than protocol-driven, potentially reducing hospital admission For paramedic: Improved decision-making and confidence to suggest patient management options that do not include hospital transfer, knowledge that can be applied to general practice visits or emergency ambulance attendances For practice and health service more widely: Broader skillset and responsibilities improve staff retention

There was agreement in the literature and interviews that rotational models were essential to avoid losing paramedics from ambulance roles; *“these posts should be rotational… because the Ambulance Trusts are haemorrhaging” (System leader, interview 5).* Yet despite the introduction of rotational roles, there were not enough paramedics to meet demand in all areas of the system (System leader, interview 6).

*“Right now I have no idea whether moving more paramedics into primary care, or taking all of the paramedics out of primary care and putting them back on the DCA [double crewed ambulance] is the right route… how do you get deployment right in a system that is in meltdown?” (System leader, interview 7).*

The logistics of employing paramedics via the rotational model appeared administratively more complex and time-consuming [32] than substantive employment and a source of risk for general practice:

*“there’s the contractual stuff around that [the rotational model], which kind of puts people off… the Practice Managers, suddenly getting a one hundred and sixty-page, national contract to have a member of staff, that’s quite scary and they’re not used to it …” [System leader, interview 6].*

Paramedics reported the experience of working across general practice and ambulance roles brought both benefits and challenges. Being employed in general practice for shorter periods of time inhibited the development of relationships between paramedics and general practice staff and made it harder to learn local systems and protocols [32]. Maintaining competencies and training across two settings was complex for paramedics [32] which had implications for retention [Social media, 2021]. There were potential risks for general practices if they invested in the development and accreditation of rotating paramedic staff who then moved on to a different organisation offering a higher band or salary [32]. However, paramedics who maintained both roles were reported to benefit from clinical development, support and improved shift patterns of general practice whilst also retaining the sense of a paramedic identity from working in urgent care (System leader interviews 1,2, 3 and 5).

## Discussion

This realist synthesis explored the role of the paramedic in UK general practice using a variety of empirical, grey and primary data sources to develop initial programme theories. The data highlighted that although the role of the paramedic in UK general practice has been introduced over a period of 20 years, it is still developing and there is a lack of clarity, for general practices, paramedics and patients, about what the role involves. This may lead to paramedics inadvertently working outside of their scope or requiring extensive supervision when transitioning into the role to ensure safe practice. Appropriate levels of support and professional development were important to help paramedics switch from ambulance to general practice settings, embed the role in practices and ensure paramedic satisfaction. Patients were generally accepting of the role, though they expressed uncertainty about who they were being seen by and whether the paramedic skillset was appropriate to general practice. The variable models of work and employment for paramedics had implications for how these roles were maintained across ambulance and general practice settings, and how the role worked for practices, paramedics and patients.

When employing a paramedic in general practice, role clarity has been highlighted as a key area of importance [44–46]. This research provides insights into the range of skillsets amongst paramedics entering general practice; not all paramedics in general practice are advanced paramedics. Historically, the wide variety of terms used to describe the role might have contributed to the lack of clarity in general practice and public understanding of the paramedic skillset. More recently the College of Paramedics has differentiated the terms ‘Specialist paramedic’ and ‘Advanced paramedic’ to refer to practitioners working at post-graduate diploma or a Masters level respectively [45]. Health Education England commissioned the College of Paramedics to detail the core clinical skills and presentations that an advanced paramedic is expected to manage [44]. Implementation guidance tends to put the onus on the paramedic to share their level of competency with general practice, but our review demonstrates that this may be challenging for paramedics working in a new setting with a new patient population, as they may have limited awareness of the range of clinical situations they may encounter.

Expectations and perceptions of the role may differ between general practice staff and paramedics resulting in dissatisfaction for both groups. Expectations need to be accurate to enable effective collaboration and to ensure appropriate supervision, and to match paramedics to appropriate patient groups. Working closely with general practice teams to test the boundaries of paramedic scope of practice across an array of presentations may be key to embedding the role successfully. As the roadmap to paramedic practice [47] becomes more embedded, and as paramedic and other first contact practitioner roles become more established in primary care, it is likely that general practice teams will become more aware of the distinctions between different paramedic skillsets and what this means for collaborative working and patient safety. However, in this interim period practice staff may require additional support to ensure appropriate understanding of paramedic skills and how to utilise these to ensure safe care and optimal practice.

Appropriate supervision of paramedics as they develop and become embedded in general practice was considered fundamental to the success of the role. The need for quality supervision is becoming more widely recognised; paramedics are advised to be guided by a named physician, particularly when completing certain advanced practice modules [45]. However, the supervision workload on GPs or other advanced practice staff is difficult to quantify. It is important to understand how the GP role is evolving, considering their ongoing responsibility for patient care, supervision of multiple allied health professional roles, and the high workload for GPs in the NHS. It might be that outsourcing paramedic supervision to educational institutions could relieve some of this burden on GPs and other practice staff.

Rotational models of employment may appear to be the solution to ensuring the paramedic workforce is not permanently displaced from ambulance trusts to primary care, and for achieving role variation and professional development that retains paramedic staff in post. However, the longer-term consequences of rotational working require further attention. For example, it is not clear how different shift patterns across primary and secondary services impact on paramedic integration into general practice teams and if this in turn may influence role satisfaction, professional development or patient outcomes [32]. Communication and collaboration with colleagues are considered benchmarks of multidisciplinary working which improve patient care [44], and inconsistent or temporary working patterns are likely to disrupt these processes. Practices may also be unclear about the benefits or challenges associated with employing paramedics directly or outsourcing this responsibility to PCNs or ambulance trusts. Each of these models will affect the practice administrative burden (e.g. training, employment processes, covering absence, indemnity issues and costs) in different ways. Understanding these models is vital to explain what makes the role successful in different contexts.

### Strengths and limitations

This rapid realist synthesis was conducted by a multidisciplinary research team, including researchers, academics, GPs, ambulance paramedics and paramedics working in general practice, and utilised public consultation at all stages. It considered a variety of data including empirical literature, interviews and social media sources, and included a wide range of stakeholder perspectives.

In accordance with realist methodology, data were not appraised or weighted based on hierarchies of methodology or source, but selected based on the relevance, rigour and richness of detail to address the research question. It was examined consistently for content to understand the point being communicated and what this might reveal about how the paramedic role works in general practice. Furthermore, as outlined in the methods section, subjective reasoning was used to theorise how context and mechanisms lead to a variety of outcomes. Consequently, the theories put forward should be treated cautiously at this stage and they will be tested empirically in the next stage of work.

## Conclusions

There is significant variation in the ways in which paramedics are working and becoming embedded in general practice settings across England. Furthermore, variation in paramedic skillsets and development requirements when entering general practice mean it is often difficult to determine how paramedics fit best into the workforce, and which patients and conditions they should manage. The understanding of the role by general practice staff does not always reflect what can be safely and efficiently delivered; equally, paramedics moving into general practice experience a sudden shift in expectations around their role, which may prove undesirable for some. Lack of clarity regarding the paramedic role may be compounded by variation in role titles and the novelty of the role in general practice; this is likely to improve as paramedics become embedded and normalised into teams over time. Rotational models of employment may bring practical benefits to paramedics and patients, but appear to be more complicated for general practice to operationalise, and may counter the advantages afforded by embedding paramedics into practice teams longer-term. The theories presented in this paper are derived from review and consultation activity and should be refined and tested in future prospective and observational studies.

### Supplementary Information

**Additional file 1: **Empirical literature search terms.

**Additional file 2: **Grey literature search parameters.

**Additional file 3: **System Leader/Key Informant Interview Topic Guide.

**Additional file 4: **Included empirical literature n=32.

**Additional file 5: **Schematic of themes.

## Data Availability

The datasets used and/or analysed during the current study are available from the corresponding author on reasonable request.

## Abbreviation

PCN: Primary Care Network

## References

[1] Hobbs FR, Bankhead C, Mukhtar T, Stevens S, Perera-Salazar R, Holt T (2016). Clinical workload in UK primary care: a retrospective analysis of 100 million consultations in England, 2007–14. The Lancet.

[2] Baird B, Charles A, Honeyman M, Maguire D, Das P (2016). Understanding pressures in general practice: King's Fund London.

[3] NHS Digital. Appointments in General Practice, October 2018. https://digital.nhs.uk/data-and-information/publications/statistical/appointments-in-general-practice/oct-2018 [Accessed 06–09–2020]

[4] NHS England. General practice forward view. 2016. https://www.england.nhs.uk/wp-content/uploads/2016/04/gpfv.pdf [Accessed 04–09–2020].

[5] NHS Digital. General Practice Workforce 30 June 2020. Available at: https://digital.nhs.uk/data-and-information/publications/statistical/general-and-personal-medical-services/30-june-2020 [Accessed 06–09–2020]

[6] PULSE. Revealed: 450 GP surgeries have closed in the last five years. 2019; Available at: http://www.pulsetoday.co.uk/news/hot-topics/stop-practice-closures/revealed-450-gp-surgeries-have-closed-in-the-last-five-years/20036793.article [Accessed 04–09–2020].

[7] NHS. The NHS long term plan. 2019; Available at: www.longtermplan.nhs.uk [Accessed 04–09–2020].

[8] McDermott, I., Spooner, S., Goff, M., Gibson, J., Dalgarno, E., Francetic, I., Hann, M., Hodgson, D., McBride, A., Checkland, K. and Sutton, M., 2022. Scale, scope and impact of skill mix change in primary care in England: a mixed-methods study. 10.3310/YWTU6690. 35593786

[9] Schofield B, Voss S, Proctor A, Benger J, Coates D, Kirby K (2020). Exploring how paramedics are deployed in general practice and the perceived benefits and drawbacks: a mixed-methods scoping study. BJGP Open..

[10] Turner J, Williams J. An Evaluation of early stage development of rotating paramedic model pilot sites. 2018; Available at: https://www.hee.nhs.uk/sites/default/files/documents/Feasability%20Study%20of%20the%20Rotating%20Paramedics%20Pilot%20-%20Final.pdf [Accessed 04–09–2020].

[11] Eaton G, Wong G, Williams V, Roberts N, Mahtani KR (2020). Contribution of paramedics in primary and urgent care: a systematic review. Br J Gen Practice.

[12] Proctor A (2019). Home visits from paramedic practitioners in general practice: patient perceptions. Journal of Paramedic Practice.

[13] Moule P, Clompus S, Lockyer L, Coates D, Ryan K (2018). Preparing non-medical clinicians to deliver GP out-of-hours services: lessons learned from an innovative approach. Educ Prim Care.

[14] Woollard M. The role of the paramedic practitioner in the UK. Australas J Paramedicine. 2015;4(1):1–9.

[15] Rasku T, Kaunonen M, Thyer E, Paavilainen E, Joronen K (2019). The core components of Community Paramedicine–integrated care in primary care setting: a scoping review. Scand J Caring Sci.

[16] Ball L (2005). Setting the scene for the paramedic in primary care: a review of the literature. Emerg Med J.

[17] Eaton G, Wong G, Tierney S, Roberts N, Williams V, Mahtani KR (2021). Understanding the role of the paramedic in primary care: a realist review. BMC Med.

[18] Jagosh J (2019). Realist synthesis for public health: building an ontologically deep understanding of how programs work, for whom, and in which contexts. Annu Rev Public Health.

[19] Pawson R, Tilley N (1997). Realistic Evaluation.

[20] Dalkin S, Greenhalgh J, Jones D, Cunningham B, Lhussier M. What’s in a Mechanism? Development of a key concept in realist evaluation. Implement Sci. (2015);10(1):1–7. 10.1186/s13012-015-0237-x PMC4408605 25885787

[21] Saul JE, Willis CD, Bitz J, Best A (2013). A time-responsive tool for informing policy making: rapid realist review. Implement Sci.

[22] Wong G, Greenhalgh T, Westhorp G, Buckingham J, Pawson R (2013). RAMESES publication standards: realist syntheses. BMC Med.

[23] College of Paramedics, 2021. The journey of the college. [cited 2021 Feb 27]. Available from: https://collegeofparamedics.co.uk/COP/About_Us/The_Journey_of_the_College.aspx

[24] Malterud K, Siersma VD, Guassora AD (2016). Sample size in qualitative interview studies: guided by information power. Qual Health Res.

[25] Jagosh, J: personal communication 2021.

[26] Abrams R, Wong G, Mahtani KR, Tierney S, Boylan AM, Roberts N, Park S (2020). Delegating home visits in general practice: a realist review on the impact on GP workload and patient care. Br J Gen Pract.

[27] Mahtani KR, Eaton G, Catterall M, Ridley A (2018). Setting the scene for paramedics in general practice: what can we expect?. J R Soc Med.

[28] Booker M, Voss S (2019). Models of paramedic involvement in general practice. Br J Gen Pract.

[29] O’Hara R, Johnson M, Siriwardena AN, Weyman A, Turner J, Shaw D, Mortimer P, Newman C, Hirst E, Storey M, Mason S (2015). A qualitative study of systemic influences on paramedic decision making: care transitions and patient safety. J health serv res policy..

[30] A ‘how-to’ guide: recruitment and development of paramedics in primary care https://gpexcellencegm.org.uk/wp-content/uploads/Recruitment-and-Development-of-Paramedics-in-Primary-Care.pdf

[31] Spence D (2017). Bad medicine: good medicine—the GP paramedic. Br J Gen Pract.

[32] Jeanes and Hamilton, 2018. Making best use of paramedics to support a sustainable urgent care and emergency care system. https://glosprimarycare.co.uk/making-best-use-of-paramedics-to-support-a-sustainable-urgent-and-emergency-care-system

[33] Royal College of General Practitioners 2015, Continuity of care in modern day general practice. Available from: https://www.rcgp.org.uk/getmedia/11f26527-5d11-47f2-a593-1a894c2fff1b/Continuity-of-care-in-modern-day-general-practice1.pdf.

[34] NHS England, No Date, Improving access with an urgent care team at Beacon Medical Group, South. https://www.england.nhs.uk/gp/case-studies/beacon-medical-group-2/

[35] Leeds Paramedic Primary Care Rotation initiative, NHS, 2018, https://aace.org.uk/initiatives/leeds-paramedic-primary-rotation/

[36] Eaton G, Happs I, Tanner R (2021). Designing and implementing an educational framework for advanced paramedic practitioners rotating into primary care in North Wales. Educ Prim Care.

[37] The Guardian, 2021. The paramedic will see you now: home visits by ambulance staff lighten GPs’ load. https://www.theguardian.com/society/2021/nov/14/the-paramedic-will-see-you-now-home-visits-by-ambulance-staff-lighten-gps-load

[38] Pulse Today, 2015. How employing a paramedic solved our recruitment problem (Beacon Medical Group). https://www.pulsetoday.co.uk/resource/workforce/how-employing-a-paramedic-solved-our-recruitment-problem/

[39] Daly J (2012). The paramedic in the community: my story. Primary Health Care.

[40] Salisbury H (2021). Helen Salisbury: No room for growth at general practices. BMJ..

[41] Barker RO, Stocker R, Russell S, Hanratty B (2021). Future-proofing the primary care workforce: A qualitative study of home visits by emergency care practitioners in the UK. European Journal of General Practice.

[42] Creton D, Halter M, LaTrobe C. PP33 A service evaluation of the experiences of specialist paramedics working in rotational environments using a retrospective cohort. Emergency Medicine Journal. 2020;37(10):e:16.

[43] Wagstaff B, Mistry V (2020). The integration of paramedics into primary care. Br J Gen Pract.

[44] College of Paramedics, 2018, Paramedic Specialist in Primary and Urgent Care Core Capabilities Framework. https://www.hee.nhs.uk/sites/default/files/documents/Paramedic%20Specialist%20in%20Primary%20and%20Urgent%20Care%20Core%20Capabilities%20Framework.pdf

[45] College of Paramedics, 2019, Employers’ Guide Paramedics in Primary and Urgent Care, https://frimleytraininghub.co.uk/wpcms/wp-content/uploads/2020/12/CoP_Employers_Guide_PUC_310719.pdf

[46] CCG: Fareham and Gosport and South Eastern Hampshire Clinical Commissioning Groups, 2018, A Guide for General Practice Employing a Paramedic: 2^nd^ Edition. https://www.oxfordhealth.nhs.uk/library/wp-content/uploads/sites/3/2018/06/2018-06-05-Employing-a-Paramedic-in-Primary-Care-Toolkit-v4.pdf

[47] HEE: Health Education England, 2021, First Contact Practitioners and Advanced Practitioners in Primary Care: (Paramedic) A Roadmap to Practice https://www.hee.nhs.uk/sites/default/files/documents/Paramedics-FINAL%20%28002%29.pdf

