# Supplementary material for: Understanding paramedic work in general practice in the UK: a rapid realist synthesis
Source: BMC Prim Care. 2024 Jan 23;25:32. doi: 10.1186/s12875-024-02271-1 (PMC10804758; doi:10.1186/s12875-024-02271-1)
Supplement: Supplementary file 1 — Additional file 1: Empirical literature search terms. [file 12875_2024_2271_MOESM1_ESM.docx]

*Additional file 1: Empirical literature search terms*

“GP*” OR “general practitioner*” OR “family practitioner*” OR “family physician*” OR “family doctor*” OR “primary care” OR “primary healthcare” OR “primary health care” OR “primary practice*” OR “general practice*” OR “family practice*”

AND

“paramedic*” OR “emergency care practitioner*” OR “urgent care practitioner*” OR “first contact practitioner*” OR “advanced practitioner*” OR "FCP"
