# Supplementary material for: Understanding paramedic work in general practice in the UK: a rapid realist synthesis
Source: BMC Prim Care. 2024 Jan 23;25:32. doi: 10.1186/s12875-024-02271-1 (PMC10804758; doi:10.1186/s12875-024-02271-1)
Supplement: Supplementary file 2 — Additional file 2: Grey literature search parameters. [file 12875_2024_2271_MOESM2_ESM.docx]

*Additional file 2: Grey literature search parameters*

| No. | Source | Parameters of search | Extracted hits |
| --- | --- | --- | --- |
| 1 | Social media data  Relevant paramedic discussion pages, and hashtags | Last 6 months (1.7.21-8.12.21) | 38 out of unknown |
| 2 | YouTube | 12 months  (1.12.20-8.12.21) | 2 out of 16 |
| 3 | Google | First 5 pages of results | 9 out of 50 |
| 4 | British Medical Association | 4 years (1.1.2017-13.12.2021) | 3 out of 36 |
| 5 | The College of Paramedics | No limits | 0 out of 30 |
| 6 | Health Education England | No limits | 6 out of 45 |
| 7 | Royal College of General Practitioners | No limits | 10 out of 18 |
| 9 | The Health and Care Professions Council | No limits | 7 out of 18 |
| 10 | Pulse Today (GP news site) | No limits | 8 out of 19 |
| 11 | Nhs.jobs | No limits | 23 out of 199 |
