# Supplementary material for: Understanding paramedic work in general practice in the UK: a rapid realist synthesis
Source: BMC Prim Care. 2024 Jan 23;25:32. doi: 10.1186/s12875-024-02271-1 (PMC10804758; doi:10.1186/s12875-024-02271-1)
Supplement: Supplementary file 3 — Additional file 3: System Leader/Key Informant Interview Topic Guide. [file 12875_2024_2271_MOESM3_ESM.docx]

*Additional file 3: System Leader/Key Informant Interview Topic Guide*

1. How does your role relate to paramedics working in primary care services?
2. What you think about the service development of having paramedics working in general practice?
3. As part of some previous research into the role we have come up with 3 ideas about how the role of paramedics in primary care may impact service delivery. It would be helpful to hear your thoughts on these.
   1. Paramedics free up GPs so they can concentrate on most complex cases
   2. Paramedics create additional capacity to manage same-day urgent problems
   3. Paramedics may improve clinical / satisfaction outcomes
4. We think that different local issues might create different models of paramedics working in primary care. Is this something you have any thoughts on? Are some models better than others?
5. What contextual factors determine the extent to which paramedics are deployed?
6. Why do you think the move to primary care has happened from a paramedic perspective?
7. What are your thoughts on the paramedic scope of practice within primary care?
8. When exploring the effectiveness of the role of the paramedic in primary care, what do you consider as ‘valued outcomes’?
