# Supplementary material for: Understanding paramedic work in general practice in the UK: a rapid realist synthesis
Source: BMC Prim Care. 2024 Jan 23;25:32. doi: 10.1186/s12875-024-02271-1 (PMC10804758; doi:10.1186/s12875-024-02271-1)
Supplement: Supplementary file 4 — Additional file 4: Included empirical literature n=32. [file 12875_2024_2271_MOESM4_ESM.docx]

*Additional file 4: Included empirical literature n=32*

| No. | Reference  * Papers reviewed by the PPI contributors |
| --- | --- |
| 1 | Barker, R.O., Stocker, R., Russell, S. and Hanratty, B., 2021. Future-proofing the primary care workforce: A qualitative study of home visits by emergency care practitioners in the UK. *European Journal of General Practice*, *27*(1), pp.68-76. |
| 2* | Booker, M. and Voss, S., 2019. Models of paramedic involvement in general practice. *British Journal of General Practice*, *69*(687), pp.477-478. |
| 3* | Brown, P., 2017. A day in the life of a paramedic advanced clinical practitioner in primary care. *Journal of Paramedic Practice*, *9*(9), pp.378-386. |
| 4 | Bulger, J., Driscoll, T., Hussain, A., Edwards, A., Evans, B., Griffiths, L., James, M., Keen, L., Kingston, M., Mclean, G. and Phillips, C., 2019. PP17 Ambulance paramedics responding to urgent patient requests in general practice for home visits–evaluation development (ARRIVE). *Emergency Medicine Journal*, 36(10), e:8 |
| 5 | Cave, J., 2018. Paramedics: what can we expect. *Journal of the Royal Society of Medicine*, *111*(11), pp.388-389. |
| 6 | Chapman, V. and Lewis, G. (2003). First contact care: a practice perspective. *Primary Health Care, 13*(10), pp.20 |
| 7 | Creton, D., Halter, M. and LaTrobe, C., 2020. PP33 A service evaluation of the experiences of specialist paramedics working in rotational environments using a retrospective cohort. *Emergency Medicine Journal, 37*(10), e:16 |
| 8 | Daly, J., 2012. The paramedic in the community: my story. *Primary Health Care, 22*(9), pp.16-19 |
| 9 | Dean, E., 2018. Greater paramedic prescribing will improve care. *Emergency Nurse*, *26*(1), pp.10-11 |
| 10 | Dixon, M., 2021. Non-medical prescribing for paramedics in primary care. *Journal of Paramedic Practice*, *13*(4), pp.140-143. |
| 11* | Dixon, M., 2020. The developing role of the paramedic prescriber. *Journal of Prescribing Practice*, *2*(2), pp.98-101. |
| 12 | Duffin, C. (2004). First in line. *Nursing Standard, 18*(18), pp.12 |
| 13 | Eaton, G., Happs, I. and Tanner, R., 2021. Designing and implementing an educational framework for advanced paramedic practitioners rotating into primary care in North Wales. *Education for Primary Care*, 32(5), pp.289-295 |
| 14 | Eaton, G., Mahtani, K. and Catterall, M., 2018. The evolving role of paramedics–a NICE problem to have? *Journal of health services research & policy*, *23*(3), pp.193-195. |
| 15 | Eaton, G., Williams, V., Wong, G., Roberts, N. and Mahtani, K.R., 2019. Protocol for the impact of paramedics in NHS primary care: application of realist approaches to improve understanding and support intelligent policy and future workforce planning. *British Paramedic Journal*, *4*(3), pp.35-42 |
| 16 | Eaton, G., Wong, G., Tierney, S., Roberts, N., Williams, V. and Mahtani, K.R., 2021. Understanding the role of the paramedic in primary care: a realist review. *BMC medicine*, *19*(1), 145 |
| 17 | Eaton, G., Wong, G., Williams, V., Roberts, N. and Mahtani, K.R., 2020. Contribution of paramedics in primary and urgent care: a systematic review. *British Journal of General Practice*, *70*(695), e.421-e426 |
| 18 | Edwards, J., Coward, M. and Carey, N., 2020. Paramedic independent prescribing in primary care: seven steps to success. *Journal of Prescribing Practice*, *2*(6), pp.292-299. |
| 19 | Jones, J., Kingston, M., Driscoll, T., Edwards, A., Evans, B., Hussain, A., James, M., Griffiths, L., Mclean, G., Keen, L. and Phillips, C., 2019. ARRIVE: Ambulance paramedics Responding to urgent patient Requests In general practice for home Visits—Evaluation development. *British Journal of General Practice*, *69*(suppl 1). |
| 20 | Mahtani, K.R., Eaton, G., Catterall, M. and Ridley, A., 2018. Setting the scene for paramedics in general practice: what can we expect?. *Journal of the Royal Society of Medicine*, *111*(6), pp.195-198. |
| 21 | Porter, A., Driscoll, T., Edwards, A., Evans, B., Griffiths, L., Hussain, A., Mari, J., Jones, J., Keen, L., Kingston, M. and Phillips, C., 2020. PP29 The paramedic will see you now: understanding new roles in primary care. *Emergency Medicine Journal: EMJ*, *37*(10), e.13 |
| 22* | Proctor, A., 2019. Home visits from paramedic practitioners in general practice: patient perceptions. *Journal of Paramedic Practice*, *11*(3), pp.115-121. |
| 23 | Robertson, D., Baines, B., Nosworthy, G., Thomas, W., Timmins, M., Watson, R., Wright, S. (2020). PP35 Evaluation of a rotational model of advanced paramedic practice in North Wales: a logic model approach to demonstrate effectiveness. *Emergency Medicine Journal. 37*(10), e.16-17. |
| 24 | Salisbury, H., 2021. Helen Salisbury: No room for growth at general practices. *British Medical Journal*, *374*, n.1691 |
| 25 | Schofield, B., Voss, S., Proctor, A., Benger, J., Coates, D., Kirby, K., Purdy, S. and Booker, M., 2020. Exploring how paramedics are deployed in general practice and the perceived benefits and drawbacks: a mixed-methods scoping study. *BJGP open*, *4*(2). |
| 26* | Spence, D., 2017. Bad medicine: good medicine—the GP paramedic. *British Journal of General Practice*, *67*(660), pp.314-314. |
| 27 | Spence, D., 2019. Bad Medicine: Advanced practitioners versus doctors. *The British Journal of General Practice*, *69*(681), p.199. |
| 28 | Stenner, K., van Even, S. and Collen, A., 2019. Early adopters of paramedic prescribing: a qualitative study. *British Paramedic Journal*, *4*(3), p.57. |
| 29 | Stenner, K., van Even, S. and Collen, A., 2021. Paramedic independent prescribing: a qualitative study of early adopters in the UK. *British Paramedic Journal*, *6*(1), pp.30-37. |
| 30 | Stones, A.J., 2019. The ACP–GP relationship. *British Journal of General Practice*, *69*(684), pp.348 |
| 31 | Wagstaff, B. and Mistry, V., 2020. The integration of paramedics into primary care. *British Journal of General Practice*, *70*(692), pp.123 |
| 32 | Woollard, M., 2006. The role of the paramedic practitioner in the UK. *Australasian Journal of Paramedicine*, *4*(1) |

Transferable Findings

| No. | Reference |
| --- | --- |
| 1 | Abrams, R., Wong, G., Mahtani, K.R., Tierney, S., Boylan, A.M., Roberts, N. and Park, S., 2018. Understanding the impact of delegated home visiting services accessed via general practice by community-dwelling patients: a realist review protocol. *BMJ open*, *8*(11), p.e024876. |
| 2 | Abrams, R., Wong, G., Mahtani, K.R., Tierney, S., Boylan, A.M., Roberts, N. and Park, S., 2020. Delegating home visits in general practice: a realist review on the impact on GP workload and patient care. *British Journal of General Practice*, *70*(695), pp.e412-e420. |
| 3 | Anthony BF, Surgey A, Hiscock J, Williams NH, Charles JM. General medical services by non-medical health professionals: a systematic quantitative review of economic evaluations in primary care. British Journal of General Practice. 2019 May 1;69(682):e304-13. |
| 4 | Hill H, McMeekin P, Price C. A systematic review of the activity and impact of emergency care practioners in the NHS. Emergency Medicine Journal. 2014 Oct 1;31(10):853-60. |
| 5 | Lyness E, Parker J, Willcox ML, Dambha-Miller H. Experiences of out-of-hours task-shifting from GPs: a systematic review of qualitative studies. BJGPopen. 2021 Aug 1;5(4). |
| 6 | Morris L, Moule P, Pearson J, Foster D, Walsh N. Patient view of the advanced practitioner (AP) role in primary care: A realist‐informed synthesis. Musculoskeletal Care. 2021 Dec;19(4):462-72. |
| 7 | Nelson PA, Bradley F, Martindale AM, McBride A, Hodgson D. Skill-mix change in general practice: a qualitative comparison of three ‘new’non-medical roles in English primary care. British Journal of General Practice. 2019 Jul 1;69(684):e489-98. |
| 8 | Officer T, Cumming J, McBride-Henry K. Successfully developing advanced practitioner roles: policy and practice mechanisms. Journal of Health Organization and Management. 2018 Oct 30. |
| 9 | Rasku T, Kaunonen M, Thyer E, Paavilainen E, Joronen K. The core components of community paramedicine–integrated care in primary care setting: a scoping review. Scandinavian journal of caring sciences. 2019 Sep;33(3):508-21. |
| 10 | Ruston, A. and Tavabie, A., 2011. An evaluation of a training placement in general practice for paramedic practitioner students: improving patient-centred care through greater interprofessional understanding and supporting the development of autonomous practitioners. *Quality in Primary Care*, *19*(3), pp.167-173. |
| 11 | Silverston P. Advanced practitioners: friend or foe?. British Journal of General Practice. 2019 Oct 1;69(687):501-. |
