# Supplementary material for: Understanding paramedic work in general practice in the UK: a rapid realist synthesis
Source: BMC Prim Care. 2024 Jan 23;25:32. doi: 10.1186/s12875-024-02271-1 (PMC10804758; doi:10.1186/s12875-024-02271-1)
Supplement: Supplementary file 5 — Additional file 5: Schematic of themes. [file 12875_2024_2271_MOESM5_ESM.docx]

*Additional file 5: Schematic of themes*

| **Overarching theme headings** | **Themes inductively generated during data extraction process** | **Data sources which elucidated themes** | **Provisional CMOcs** |
| --- | --- | --- | --- |
